# A left-sided cystic pancreatic incidentaloma with sigmoid colon adenocarcinoma: a case report

**DOI:** 10.1186/s13256-018-1778-9

**Published:** 2018-08-30

**Authors:** P. J. Halder, Swapnil Sharma, Nikhil S

**Affiliations:** 1 Department of Surgical Gastroenterology and HPB Surgery, Jagjivan Ram Hospital, Maratha Mandir Lane, Mumbai Central, Mumbai, 400008 India; 2 DNB Surgical Gastroenterology, Jagjivan Ram Hospital, Mumbai, 400008 India

**Keywords:** Pancreatic incidentaloma, Serous cystadenoma pancreas, Sigmoid carcinoma

## Abstract

**Background:**

The synchronous colorectal malignancy is well described in the literature but combination of pancreatic incidentaloma with sigmoid cancer has not been well described and the association has not been described in syndrome.

**Case presentation:**

A 65-year-old man from the Indian subcontinent with a history of abdominal pain with loss of appetite, and with a history of bleeding per rectum and altered bowel habits presented to our hospital. An abdominal examination revealed a palpable mass in the region of his epigastrium and left hypochondrium, and a rectal examination was normal. A work-up included blood investigations, an abdominal contrast-enhanced computed tomography scan, a colonoscopy, and a positron emission tomography/computed tomography scan. He was managed by simultaneous distal pancreaticosplenectomy and radical sigmoidectomy. The final histopathology results were suggestive of moderately differentiated adenocarcinoma of the sigmoid colon with serous cystadenoma of the pancreas.

**Conclusions:**

The synchronous sigmoid colon cancer and pancreatic cystic incidentaloma is a rare presentation, which, to the best of our knowledge, has not been reported in the literature. We report the surgical management of this case and present a review of the literature. Genetic studies may be conducted to find out whether there is common genetic mutation resulting in these two malignancies, and may be helpful in screening programs.

## Background

The synchronous colorectal malignancy is well described in the literature and the incidence ranges between 2 and 9% [1]. Association of pancreatic cystic neoplasm and symptomatic colonic malignancy has not been well described in the literature. In a series of 106 patients with serous cystadenoma, Tseng *et al*. [2] found only three patients with colon adenocarcinoma (< 3%) signifying a very low association.

## Case presentation

### Patient information

A 65-year-old man from the Indian subcontinent presented to our hospital with complaints of a mild, dull, aching left-sided abdominal pain for the past year, loss of weight and appetite for 6 months. There was a history of per rectum bleeding and recent history of altered bowel habits. He had no history of malignancy in the family. There was no other clinically significant history.

#### Clinical Findings

A general examination of our patient was within normal limits. There was no significant lymphadenopathy.

On abdominal examination, a 7 cm × 6 cm size lump was palpable in the epigastric region extending up to the left hypochondrium; it was nodular, nontender, firm in consistency, with a well-defined border, and not moving with respiration. The rest of his abdomen was unremarkable. A rectal examination was normal.

### Diagnostic assessment

Routine laboratory investigations including a complete blood count, an international normalized ratio, liver function tests, and renal function tests were within normal limits. Ultrasonography of his abdomen and pelvis was performed, and a multicystic lesion in the distal body and tail of the pancreas measuring about 7 × 7 cms was seen; no lymph nodes were seen. The rest of his pancreas was normal, and the proximal pancreatic duct was dilated, his liver was normal, and no free fluid was seen. Abdominal and pelvic contrast-enhanced computed tomography (CECT) was performed and revealed a multicystic lobulated mass arising from the distal body and tail of the pancreas with proximal pancreatic duct dilatation, a normal liver, no free fluid, an irregular mass in the sigmoid colon with mild narrowing of the lumen, with no proximal dilatation of the colon. Tumor marker tests showed carcinoembryonic antigen (CEA) test results of 10.48 ng/ml (normal value. < or = 3.0 ng/mL, in smokers: < or = 5.0 ng/mL), and a CA19–9 test result of 7.19 U/mL (< 37 U/mL). A colonoscopy revealed ulceroproliferative, nonobstructing growth in the distal sigmoid colon with small polyps nearby. Colonoscopic biopsy result showed well-differentiated adenocarcinoma. A positron emission tomography/computed tomography (PET-CT) scan was performed and showed metabolically active circumferential thickening in the distal sigmoid colon with a maximum standard uptake value (SUV max) 7.0. and a multicystic lobulated mass with low metabolic activity arising from the distal body and tail of pancreas.

**Fig. 1:**
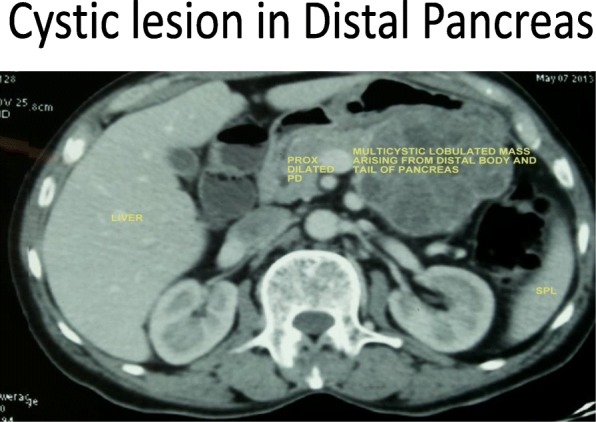
Cystic lesion in distal pancreas on computed tomography

**Fig. 2:**
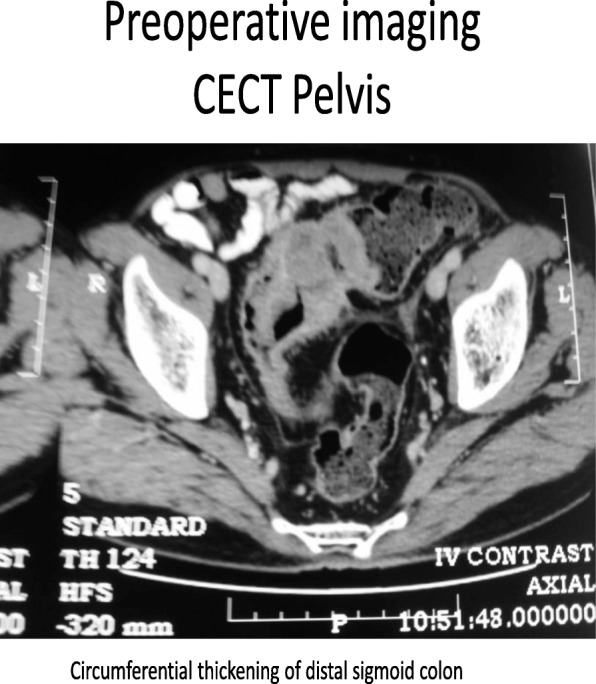
Circumferential thickening of sigmoid colon on contrast-enhanced computed tomography.

Endoscopic ultrasound confirmed the picture of microcystic serous cystadenoma with no vessel involvement or lymph nodes. An endoscopic ultrasound-guided fine-needle aspiration biopsy (EUS- FNA) was done, his fluid amylase levels were within normal limits, and his fluid CEA levels were not elevated. Cytology showed only benign cells (Figs. 1 and 2).

### Therapeutic intervention

Distal pancreaticosplenectomy and radical sigmoidectomy with end-to-end colorectal anastomosis was performed.

### Follow-up and outcome

His postoperative course was uneventful. The histopathology report showed microcystic (benign) serous cystadenoma of the pancreas, and moderately differentiated adenocarcinoma of the sigmoid colon with lymph node metastasis 0/37. Angiolymphatic invasion was positive, and perineural invasion was absent.

### Pathological staging: pT3pN0

**Fig. 3:**
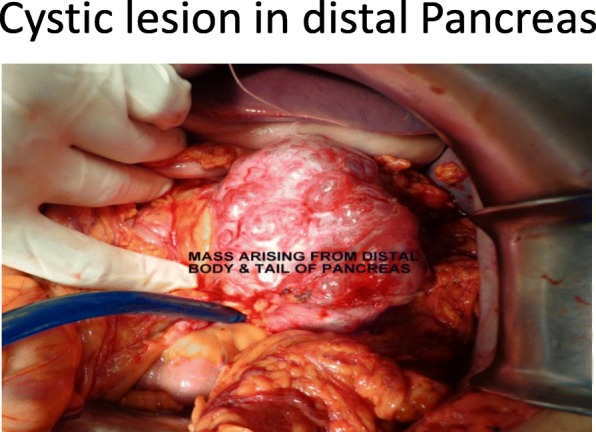
Cystic lesion in distal pancreas

**Fig. 4:**
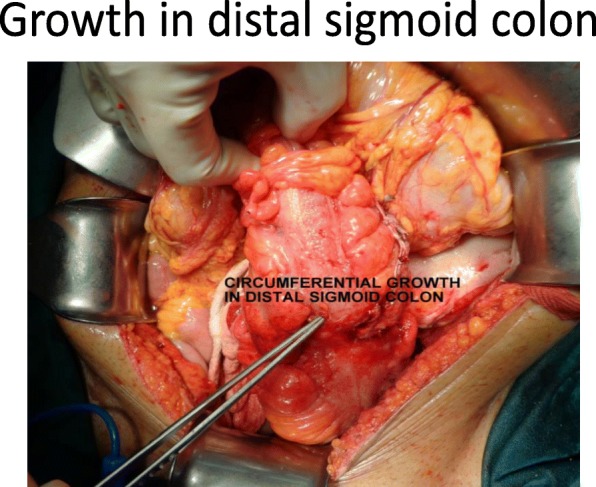
Growth in distal sigmoid colon

**Fig. 5:**
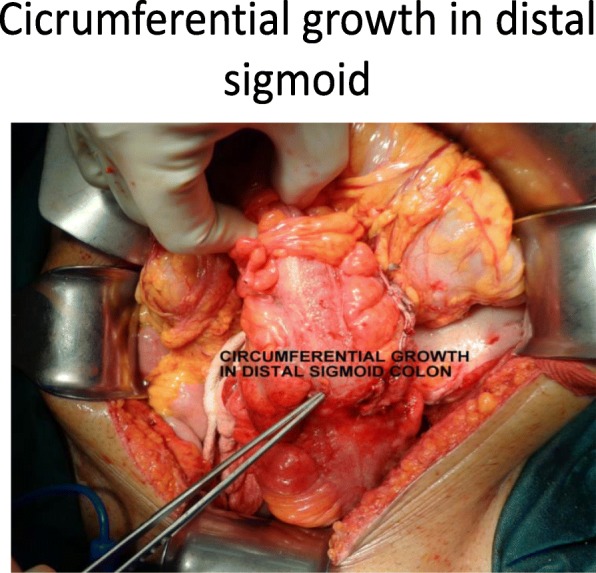
Circumferential growth in distal sigmoid

Our patient was followed up every third month for 1 year and was evaluated. Blood reports and imaging studies were unremarkable and did not detect any recurrence (Figs. 3, 4 and 5).

## Discussion

This case report deals with a rare association of a pancreatic incidentaloma (a large pancreatic serous cystadenoma) with a symptomatic sigmoid colon cancer, which was dealt by simultaneous distal pancreticosplenectomy for the pancreatic serous cystadenoma and radical sigmoidectomy for the sigmoid colon cancer. Such a combined resection, to the best of our knowledge, has not been reported in the literature. The distal pancreaticosplenectomy was completed first to facilitate and avoid a theoretical possibility of contamination of the pancreatic area with colonic contents; also the colonic resection was facilitated by resecting the large pancreatic tumor impeding the left colon mobilization. Subsequently the sigmoid resection was completed, with an end-to-end descending colon to rectum anastomosis.

Pancreatic incidentaloma (PI) has been defined as a mass that is incidentally discovered during an imaging study for symptoms other than the ones of the mass itself or the organ affected. The term was first described by Ho, *et al*. [3] and Kostiuk [4].

Left-sided cystic pancreatic incidentalomas are uncommon neoplasms, but a significant number of the patients harbor early stages or frank malignancy, hence an aggressive approach may be required [5].

Management of a cystic pancreatic incidentaloma (PI) is a complex problem as there is a lot of debate in the management of cystic lesions of the pancreas. Gore *et al*. [6] have recommended that surgery should be considered for cysts > 3 cm. size, but surgery for serous cystadenoma surgery should be deferred till the cyst is > 4 cm.

Large (> 4 cm) serous cystadenomas are more likely to be symptomatic. Although the median growth rate for this neoplasm is only 0.6 cm/year, it is significantly greater in large tumors. Whereas expectant management is reasonable in small asymptomatic tumors, resection for large serous cystadenomas is recommended, regardless of the presence or absence of symptoms [3].

Serous cystadenoma are generally considered benign cystic tumors of the pancreas. Very rarely, they are malignant (< 1% incidence) [2, 6, 7].

In a multi-institutional retrospective study of 398 patients, Le Borgne *et al*. [8] found only three cases of serous cystadenoma of pancreas in association with renal cell carcinoma and von Hippel-Lindau disease. In another series of 106 patients of serous cystadenoma, Tseng *et al*. [2] found only three patients with colon adenocarcinoma (< 3%) signifying a very low association, but the authors did not mention the treatment modality adopted in these three cases.

The role of F-18-fluorodeoxyglucose positron emission tomography (FDG-PET) has been discussed Tseng *et al*. [2] and Sperti *et al*. [9]. FDG-PET has an important role in distinguishing between benign and malignant cystic pancreatic lesions. A negative result may avoid unnecessary operations in asymptomatic or high-risk patients. In our case, a FDG-PET scan revealed a high uptake by the sigmoid tumor, but the uptake by the pancreatic tumor was low suggesting a benign tumor.

Endoscopic ultrasound is increasingly being used in characterization of cystic pancreatic lesions because it provides the highest resolution of the lesion and guided fine-needle aspiration can be done for analysis of the fluid for cytology, viscosity, amylase levels, tumor marker levels, and molecular analysis that may contribute significantly in management of the case [10].

In the literature, there is not much data on concomitant occurrence of serous cystadenoma and colorectal malignancy, but colorectal cancers are the most common extrapancreatic malignancy associated with intraductal papillary mucinous neoplasm (IPMN) in studies performed in Western patients ranging from 3 to 12% [11, 12], while gastric cancer was reported in 6–13% of Asian patients with IPMN [13, 14].

## Conclusions

This case report deals with a rare combination of a large cystic pancreatic incidentaloma, a benign serous cystadenoma, with a symptomatic sigmoid colon cancer, which was successfully managed with a simultaneous distal pancreaticosplenectomy and radical sigmoidectomy, in that sequence. To the best of our knowledge, such a surgical procedure in a case of cystic pancreatic incidentaloma has not been reported. Genetic studies may be conducted to find out whether there is common genetic mutation resulting in these two malignancy and may be helpful in screening programs.

## References

[1] Lam A, Carmichael R, Buettner PG, Gopalan V, Ho YH, *et al*. Clinicopathological significance of synchronous carcinoma in colorectal cancer. Am J Surg. 2011;202(1):39–44. 10.1016/j.amjsurg.2010.05.012 21600553

[2] Tseng JF, Warshaw AL, Sahani DV, Lauwers GL, Rattner DW, Fernandez - del Castillo C. Serous cystadenoma of the pancreas – tumor growth rates and Recommendations for treatment. Ann Surg. 2005;242(3):413–21. 10.1097/01.sla.0000179651.21193.2c PMC1357749 16135927

[3] Ho CL, Dehdashti F, Griffith LK, *et al*. FDG-PET evaluation of indeterminate pancreatic masses. J Comput Assist Tomogr. 1996;20:363–9. 10.1097/00004728-199605000-00006 8626891

[4] Kostiuk TS. Observation of pancreatic incidentaloma. Klin Khir. 2001;9:62–3. 11785413

[5] Chiarelli M, Gerosa M, Tagliabue F, *et al*. Left-sided pancreatic incidentalomas treated with laparoscopic approach: a report of 20 cases. World J Surg Oncol. 2016;14:204. 10.1186/s12957-016-0949-7 PMC4973032 27487847

[6] Gore RM, Wenzke DR, Thakrar KH, Newmark GM, Mehta UK, Berlin JW. The incidental cystic pancreatic mass: a practical approach. Cancer Imaging. 2012;12:414–21. 10.1102/1470-7330.2012.9054 PMC3460559 23022726

[7] Horvath KD, Charbot JA. An aggressive resectional approach to cystic neoplasms of the pancreas. Am J Surg. 1999;178:269–74. 10.1016/s0002-9610(99)00186-5 10587182

[8] Le Borgne J, de Calan L, Partensky C. Cystadenomas and cystadenocarcinomas of the pancreas: a multiinstitutional retrospective study of 398 cases. French Surgical Association. Ann Surg. 1999;230:152–61. 10.1097/00000658-199908000-00004 PMC1420857 10450728

[9] Sperti C, Pasquali C, Decet G, *et al*. F-18-fluorodeoxyglucose positron emission tomography in differentiating malignant from benign pancreatic cysts: a prospective study. J Gastrointest Surg. 2005;9:22–9. 10.1016/j.gassur.2004.10.002 15623441

[10] Petrone MC, Arcidiacono PG. Role of endosocopic ultrasound in the diagnosis of cystic tumours of the pancreas. Dig Liver Dis. 2008;40:847–53. 10.1016/j.dld.2008.03.019 18499542

[11] Reid-Lombardo KM, Mathis KL, Wood CM, Harmsen WS, Sarr MG. Frequency of extrapancreatic neoplasms in intraductal papillary mucinous neoplasm of the pancreas: implications for management. Ann Surg. 2010;251:64–9. 10.1097/SLA.0b013e3181b5ad1e 19858708

[12] Baumgaertner I, Corcos O, Couvelard A, Sauvanet A, Rebours V, Vullierme MP, Hentic O, Hammel P, Lévy P, Ruszniewski P. Prevalence of extrapancreatic cancers in patients with histologically proven intraductal papillary mucinous neoplasms of the pancreas: a case-control study. Am J Gastroenterol. 2008;103:2878–82. 10.1111/j.1572-0241.2008.02142.x 18853975

[13] Oh SJ, Lee SJ, Lee HY, Paik YH, Lee DK, Lee KS, Chung JB, Yu JS, Yoon DS. Extrapancreatic tumors in intraductal papillary mucinous neoplasm of the pancreas. Korean J Gastroenterol. 2009;54:162–6. 10.4166/kjg.2009.54.3.162 19844152

[14] Yoon WJ, Ryu JK, Lee JK, Woo SM, Lee SH, Park JK, Kim YT, Yoon YB. Extrapancreatic malignancies in patients with intraductal papillary mucinous neoplasm of the pancreas: prevalence, associated factors, and comparison with patients with other pancreatic cystic neoplasms. Ann Surg Oncol. 2008;15:3193–8. 10.1245/s10434-008-0143-4 18784959

